# Cross-sectional increase of adherence to multidisciplinary tumor board decisions

**DOI:** 10.1186/s12885-018-4841-4

**Published:** 2018-09-29

**Authors:** S. Hollunder, U. Herrlinger, M. Zipfel, J. Schmolders, V. Janzen, T. Thiesler, E. Güresir, A. Schröck, F. Far, T. Pietsch, D. Pantelis, D. Thomas, S. Vornholt, N. Ernstmann, T. Manser, M. Neumann, B. Funke, I. G. H. Schmidt-Wolf

**Affiliations:** 1 Department of Integrated Oncology - CIO Bonn, University Hospital Bonn, Sigmund-Freud-Strasse 25, 53105 Bonn, Germany; 2 Department of Neurooncology, Center for Integrated Oncology, University of Bonn, Bonn, Germany; 3 Department of Internal Medicine III, University Hospital Bonn, Bonn, Germany; 4 Department of Orthopedics and Trauma Surgery, University Hospital Bonn, Bonn, Germany; 5 Department of Pathology, University Hospital Bonn, Bonn, Germany; 6 Department of Neurosurgery, University Hospital Bonn, Bonn, Germany; 7 Department of Otorhinolaryngology, Head and Neck Surgery, University Hospital Bonn, Bonn, Germany; 8 Department of Oral, Maxillofacial and Plastic Surgery, University Hospital Bonn, Bonn, Germany; 9 Department of Neuropathology, University Hospital Bonn, Bonn, Germany; 10 Department of General, Visceral-, Thoracic and Vascular Surgery, University Hospital Bonn, Bonn, Germany; 11 Department of Radiology, University Hospital Bonn, Bonn, Germany; 12 Department of Psychosomatic Medicine and Psychotherapy, University Hospital Bonn, Bonn, Germany; 13 University of Applied Sciences and Arts Northwestern Switzerland, FHNW School of Applied Psychology, Bern, Switzerland

**Keywords:** Tumor board, Multidisciplinary meeting, Deviance, Adherence, Head and neck, Sarcoma and musculoskeletal tumor, Neurooncology

## Abstract

**Background:**

Cancer research has made great progress in the recent years. With the increasing number of options in diagnosis and therapy the implementation of tumorboards (TUBs) has become standard procedure in the treatment of cancer patients. Adherence tests on tumor board decisions are intended to enable quality assurance and enhancement for work in tumor boards in order to continuously optimize treatment options for cancer patients.

**Methods:**

Subject of this study was the adherence of the recommendations made in three of 14 tumorboards, which take place weekly in the Center for Integrated Oncology (CIO) at the University Hospital Bonn. In total, therapy recommendations of 3815 patient cases were checked on their implementation. A classification into four groups has been made according to the degree of implementation. A second classification followed regarding the reasons for differences between the recommendation and the therapy which the patient actually received.

**Results:**

The study showed that 80.1% of all recommendations in the three TUBs were implemented. 8.3% of all recommendations showed a deviance. Most important reasons for the deviances were patient wish (36.5%), patient death (26%) and doctoral decision, due to the patient’s comorbidities or side effects of the treatment (24.1%).Interestingly, deviance in all three tumor boards in total significantly decreased over time.

**Conclusions:**

Aim of the study was to clarify the use of tumor boards and find approaches to make them more efficient. Based on the results efficiency might be optimized by increased consideration of patients` preferences, improved presentation of patient-related data, more detailed documentation and further structuring of the tumor board meetings.

## Background

In recent years the diagnostic and therapeutic options in oncology have become much more complex not only due to advanced research but also many other developments in the field of cancer. Therefore, the interdisciplinary cooperation in the care of oncological patients has become more indispensable and has an influence on the quality of results [1].

Conditions for an interdisciplinary collaboration are written down in a certification program, which was developed by the German Cancer Society. This program requires the implementation of so called tumorboards (TUBs). These meetings, also known as multidisciplinary tumorboards (MDT) or tumor conferences, have become a standard in oncological patient care in the recent years all over the world [2–4]. These are regular meetings of medical specialists from different disciplines to evaluate a therapy recommendation, which is individual for every oncological patient presented at the TUB. It is the aim of a TUB to develop a therapy recommendation, which is evaluated in a multidisciplinary team and individual for every oncological patient because the modern way of oncological therapy cannot be monodisciplinary anymore due to the complexity of cancer [5]. In opposite to the monodisciplinary consultancy system the multidisciplinary therapy recommendation clearly provides advantages for the patient and the medical specialists [6]. The proven benefits for patients should give enough reasons for further development of the multidisciplinary performance provided by the TUBs [7].

The therapy recommendations of the TUBs are denoted as binding in the certification program of the German Cancer Society. For the adherence of the TUB-recommendations they demand that any deviance from the original recommendation must be documented and assessed. Depending on the reason for the deviances measures must be taken to avoid them (Deutsche Krebsgesellschaft).To meet these requirements of the German Cancer Society, this study made a start with the documentation of deviances and substantiated those with reasons.

Because there is no definition set for the term “deviation” in comparable studies, for this study it was defined as follows: the therapy the patient actually received deviates completely from the recommendation, which was documented in the TUB. This definition is needed because existing studies concerning the adherence of TUB decisions are always built on individual measures, which leads to a lack of comparability [8, 9].

In the Center for Integrated Oncology at the University Hospital Bonn 14 tumorboards take place weekly. Each cancerous disease can be assigned to one of those TUBs. Aim of the study was to proof the benefit of TUBs, which has been discussed in different studies [10], to display fields with potential of improvement and to find ways to optimize the work in the TUBs.

## Methods

In this study an adherence testing was performed in three of the 14 TUBs with the objective of representing three different sized tumor entities. Each tumor board consists of at least one representative of each subject area, which is involved in the patient’s treatment. These physicians meet for a panel concerning the treatment plan for patients which were announced before. By means of all diagnostic material a discussion takes place to find the best suitable treatment for each patient which is recorded in a protocol. Retrospectively, the therapeutic recommendations documented in the protocols, were tested for their implementation for each patient case which was presented at the three TUBs from June 2014 to December 2016. For this purpose, the exact wording of the protocol was checked for implementation by using different data collection portals which are used at the University Hospital Bonn. These data collecting portals gave information about all treatments the patient received in the University Hospital Bonn as well as medical reports of foreign doctors. Following those information a classification into four groups was made according to the degree of implementation:

- Group 1: the recommendation was completely implemented as recorded.
- Group 2: the recommendation was partly implemented as recorded.
- Group 3: the available data did not lead to any conclusion if or which therapy followed the recommendation given in the TUB. These recommendations are rated neither among the implemented nor deviated.
- Group 4: the actually received therapy deviated completely from the recommendation made in the TUB.

There was set a time limit of 1 year for realization of the TUB’s recommendation. According to this pattern, the patient cases were divided into the four groups. If a recommendation was not fully implemented or deviated completely, the reason for the differences was determined. There was another classification made into 8 groups according to the reason for the deviance: patient wish, patient death, doctor decision, treatment change ex domo, missing documentation, missing follow-up, outside the observation period and drug currently not available (Table 1). These 8 groups were created during the analysis of the protocols by investigating all information concerning the actually received therapy given by the data collecting portals. These were the hospital information system Orbis NICE (Agfa HealthCare GmbH, Bonn, Germany), the tumor documentation system ODSeasyNet (asthenis GmbH, Aschheim, Germany), the “Picture Archiving and Communication System” and the documentation management system HYDMedia (Agfa HealthCare GmbH, Bonn, Germany). For each patient case the therapy recommendation and the actually received therapy has been compared in a table and were assigned to one of the groups in each category.

**Table 1:** Reasons for differences between the recommended therapy and the therapy actually received

Reasons for differences	Definition of the reason
Patient wish (according to DKG-definition)	The patient refused the recommended therapy
Doctor decision (according to DKG-definition)	A change in therapy was justified by the patient’s comorbidities, general condition or occurring side effects.
Patient death	The patient died before the beginning of the recommended therapy.
Missing follow-up	Termination of therapy documentation
Treatment change ex domo	Ex domo a different therapy was chosen.
Missing documentation	Reasons for a change in therapy were unknown.
Outside the observatory period	Performance period was in the future.
Recommended drug was not available at this time	In one single case the patient had a change in therapy because the recommended drug was not available at this time.

For the data analysis the Cochran-Armitage test was performed for frequency comparison. The test was performed two-sided with a level of significance at α = 0.05 (*p* ≤ 0.05: significant result). For comparing the absolute frequencies of the investigated years the Fisher’s exact test was performed. All figures and tables were generated with Microsoft Office Excel 2007.

## Results

Subject of the study were 3815 patient cases, concerning 2450 patients, dividing up the three different entities as follows:

- ➢TUB for neurooncological tumors: 2176 patient cases, concerning 1406 patients, presented from June 2014 to December 2016 as well
- ➢TUB for head and neck tumors: 1319 patient cases, concerning 812 patients, presented from June 2014 to December 2016.
- ➢TUB for sarcomas and musculoskeletal tumors: 320 patient cases, concerning 232 patients, presented during the same period.

### Conclusion of the results in the three TUBs

**Fig. 1:**
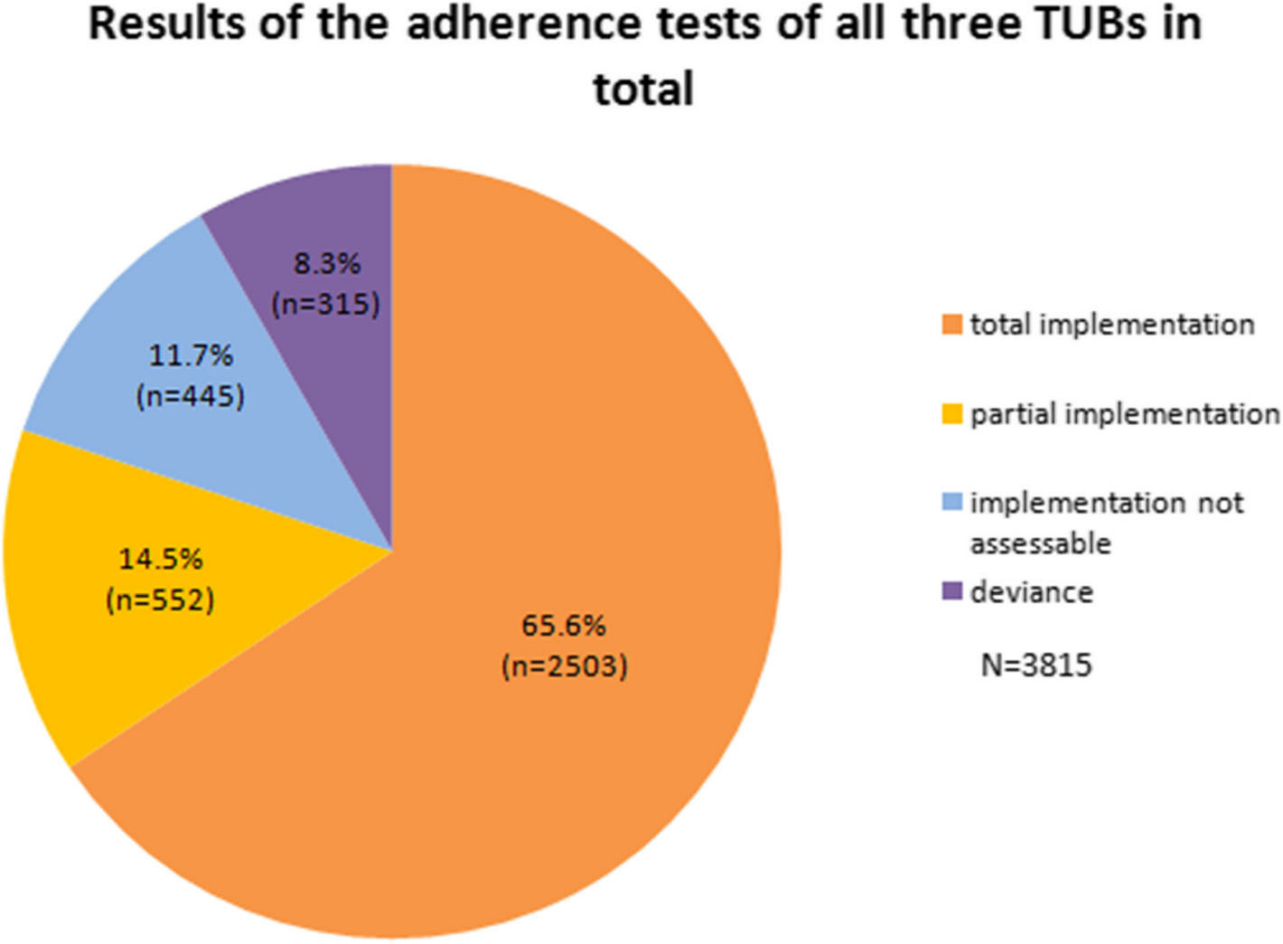
Results of the adherence tests of all three TUBs in total

In total 3815 therapy recommendations were reviewed in the three TUBs during the observed period from June 2014 till December 2016. The implementation rate was 80.1%, 65.5% was completely implemented and 14.4% was partly implemented. In 11.7% it was not assessable if the recommendation was implemented. A deviance of the actually received therapy from the recommendation was given in 8.3% (Fig. 1).The reason for the deviance was in 36.5% the patient wish, in 24.1% the doctor decision (72.4% of them are caused due to the deterioration of the patient’s general condition, comorbidities and occurring side effects; 27.6% due to inappropriate therapy recommendations due to missing data), in 26% the patient death, in 2.9% the therapy change was decided ex domo, in 10.2% the reason for the deviance was not obvious because of insufficient documentation and in 0.3% of the deviances the recommended drug was not available at this time (Table 2).

**Table 2:** Reasons for the deviances and their percentage in all of the three TUBs in total

Reasons for deviances	number	percentage
patient wish	115	36.5
doctor decision	76	24.1
patient death	82	26.0
missing follow-up	0	0.0
treatment ex domo	9	2.9
missing documentation	32	10.2
outside of the observed period	0	0.0
recommended drug not available	1	0.3
total	315	100.0

**Fig. 2:**
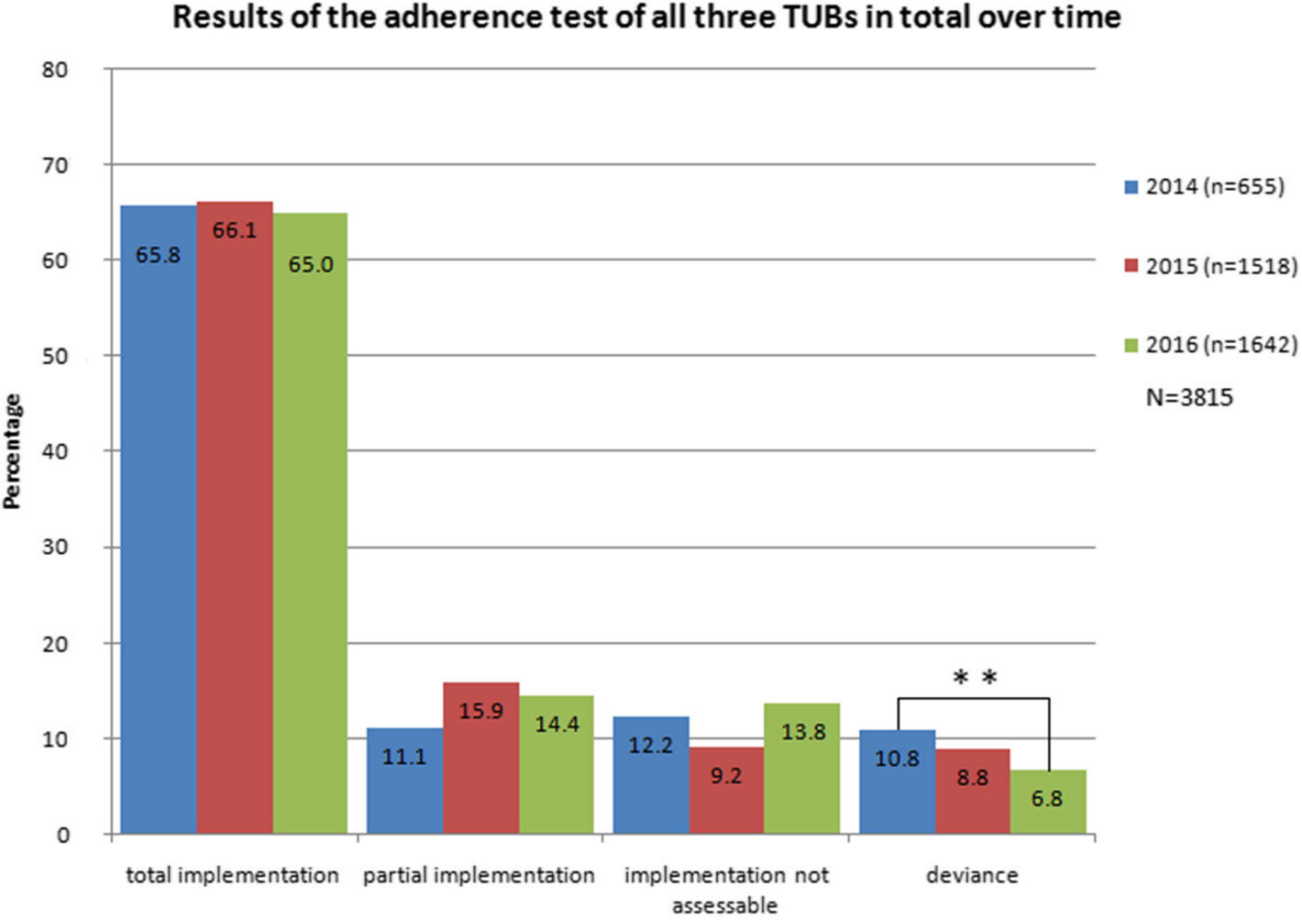
Results of the adherence test of all three TUBs in total over time (**: significant at *p* ≤ 0.01; *p* = 0.002)

Most interestingly, over time the deviance rate in summary of all three TUBs decreased from 10.2% in 2014 and 8.8% in 2015to 6.8% in 2016 (Fig. 2) (*p* = 0.002). That means a significantly decrease of the deviance rate in total. Comparing the years individually, the Fisher’s exact test showed that between the years 2014 and 2015 the deviance rate decreased not significantly (*p* = 0.089) and as well not between 2015 and 2016 (*p* = 0.089).

### TUB for neurooncological tumors

In this tumorboard a therapy recommendation was worked out for 2176 patient cases, concerning 1406 patients. 1738 of these were implemented (79.8%), 1387 completely (63.7%) and 351 partly (16.1%). In 12.5% of the cases the implementation could not be assessed (273 of the 2176 patient cases). In 165 cases (7.6%) there was a complete deviance from the recommendation given. The reason for the deviance was in 33.5% the patient wish, in 22% the doctor decision, in 34.1% the patient death, in 1.2% the deviance took place ex domo and in 8.5% the reason for deviance was not obvious because of insufficient documentation. One single case deviated because the recommended drug was not available at this time.

In the course of the years there is a deviance rate of 8.5% in 2014, 8.9% in 2015 and 6.0% in 2016 (Fig. 2) (*p* = 0.093). The individual comparison showed that the absolute frequencies of the years do not differ significantly (the Fisher’s exact test showed from 2014 to 2015: *p* = 0.914; from 2015 to 2016: *p* = 0.056; from 2014 to 2016: *p* = 0.156).

### TUB for head and neck tumors

**Fig. 3:**
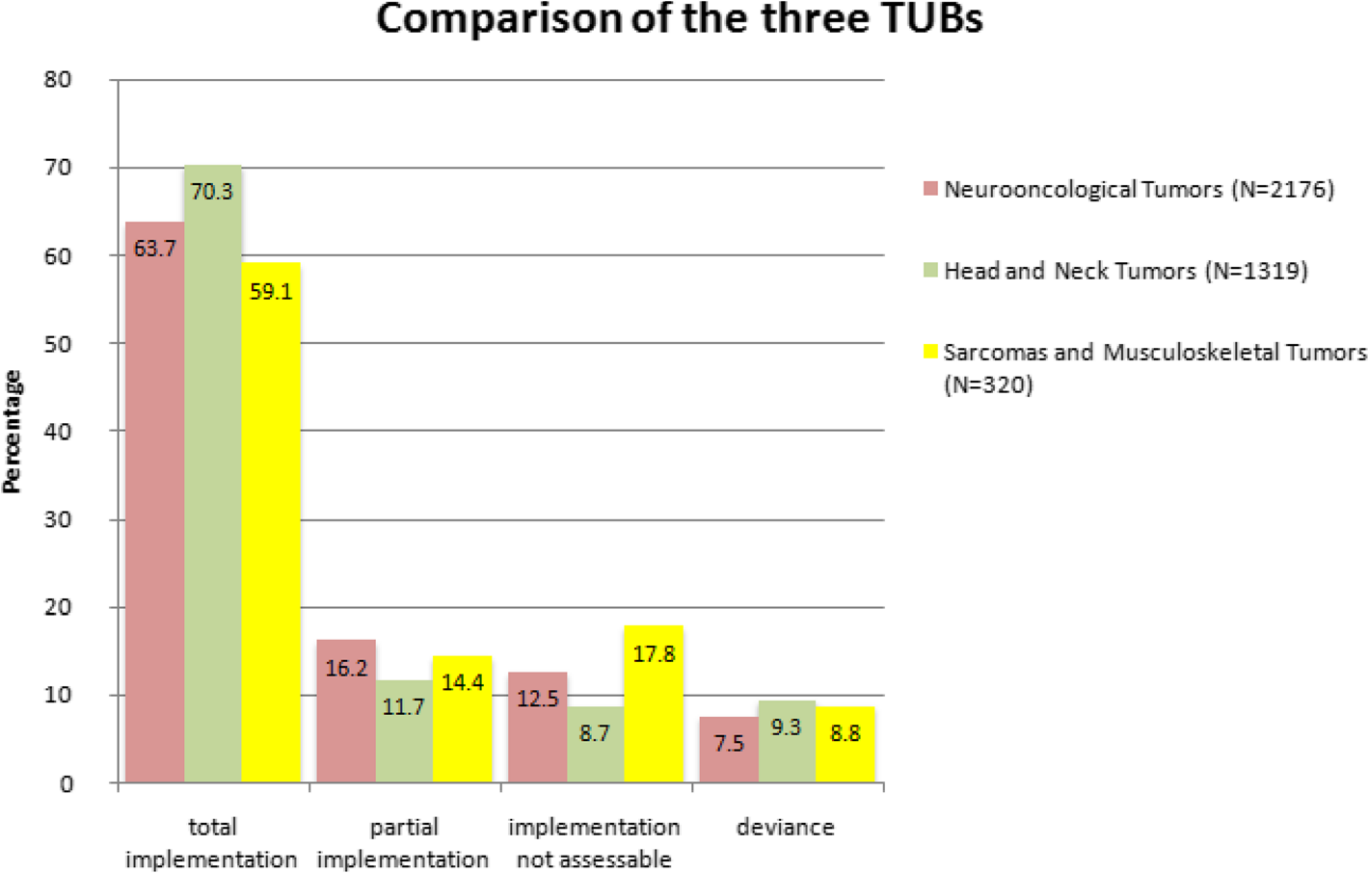
Results of the three different TUBs in comparison

In this TUB 1319 patient cases, concerning 812 patients, were discussed and therapy recommendations were worked out. 1081 of these recommendations (82%) were implemented, 927 (70.3%) completely, 154 (11.7%) partly. The implementation could not be assessed in 115 of 1319 cases (8.7%). A deviance from the recommendation to the actually received therapy was given in 123 cases (9.3%) (Fig. 3). These deviances were caused by the patient wish in 45.5%, by doctor decision in 26.8%, 17.1% of the recommendations deviated because of patient death, 2.4% of the patients with a differed therapy were treated ex domo and in 8.1% the reason for the deviance was not documented.

In the course of the years there is shown that the deviance rate goes down from 15.2% in 2014 to 8.7% in 2015 and 7.8% in 2016 (*p* = 0.003). The Fisher’s exact test showed that the deviance rate decreased significantly from 2014 to 2015 (*p* = 0.004) and from 2014 to 2016 (*p* = 0.001). Between 2015 and 2016 the decrease was not significant (*p* = 0.661).

### TUB for sarcomas and musculoskeletal tumors

Three hundred twenty patient cases, concerning 232 patients, were presented in this TUB. 235 of the documented therapy recommendations were implemented (73.5%), 189 of them completely (59.1%) and 46 partly (14.4%). The implementation of the therapy recommendation could not be assessed in 46 patient cases (17.8%). A deviance from the TUB’s recommendation was detected in 28 cases (8.8%). 14.3% of the deviances were caused by patient wish, 25% by the doctor decision established by occurring side effects, deterioration of the patient’s general condition or comorbidities, 17.9% of the deviances can be attributed to the patient’s death before the beginning of the therapy. In 14.9% of the deviances the patient got a deviant therapy ex domo and in 28.6% the reason for the deviance has not been documented.

Over time a deviance rate of 11.1% in the year 2014, 9.4% in the year 2015 and 7.6% in 2016 was observed (*p* = 0.563). The Fisher’s exact test showed that the decrease between the single years was not significant (2014/2015: *p* = 0.763; 2015/2016: *P* = 1.0; 2014/2016: *p* = 0.548).

### Comparison of the different entities

Table 3 shows the reasons for the deviances and their percentage in the three TUBs in comparison. As shown the main reasons for a deviance are the same. Only particularity is the high rate of cases with missing documentation in the TUB for sarcomas (28.6%).

**Table 3:** Reasons for the deviances and their percentage in the TUB for neurooncological tumors, head and neck tumors and sarcomas and musculoskeletal tumors

Reasons for deviances	Neurooncological TUB (%)	Head and Neck TUB (%)	Sarcomas TUB (%)
Patient wish	33.5	45.5	14.3
Doctor decision	22.0	26.8	25.0
Patient death	34.1	17.1	17.9
Missing follow-up	0.0	0.0	0.0
Treatment ex domo	1.2	2.4	14.3
Missing documentation	8.5	8.1	28.6
Outside the observed period	0.0	0.0	0.0
Recommended drug not available	0.6	0.0	0.0

Therefore, in summary it can be said, that by comparing the deviance rates of all three TUBs, they do not differ significantly (Fig. 3) (*p* = 0.125).

## Discussion

In this study the therapy recommendation in every single patient case was tested on its implementation. Therefore the TUB’s protocols were analyzed retrospectively and compared to the therapy the patient actually received. There are other comparable studies, which also performed an adherence testing. Those were constructed differently to the study at the University Hospital Bonn. A study about the Brain tumorboard (BTB) at the University Hospital Freiburg contains a collective of 500 therapy recommendations, which were checked on their implementation [8]. In that study there was a deviance rate of 9%. Deviating from the study in Bonn the adherence test in Freiburg was only performed in one TUB (the Brain Tumor Board), furthermore only patients were considered who were treated at the University Hospital Freiburg itself. Additionally statistical evaluations were performed regarding diagnosis and recommended type of treatment. Furthermore it was shown, which therapy replaced the recommendation in the case of deviance (57.8% were replaced by a watchful waiting strategy or was changed due to the patient’s general condition, 31.1% by another treatment as considered by the responsible physician). Another study from Great Britain set the deviance rate at 15% [9].The British study included patients with upper gastrointestinal cancer. It was shown that discordant decisions were more frequent for patients with pancreatic or gastric carcinoma as compared to esophageal cancer (*p* = 0.001). In the present study it was more important to compare the deviance rate in different TUBs for different tumor entities in the same hospital. Comparing those studies with our study it becomes clear that adherence testing is always based on different measures and basics and that a uniform definition of the term “deviance” is needed.

This point leads to limitations which had influence on the present study. A so far missing definition of the term “deviance” entails differences in the evaluation. In the present study the 8 groups of reasons for a deviance were introduced by one person with the ambition to give as much detailed information as possible. This automatically results in subjective assessment in some fields of the study. For further development of the TUBs on an international level a uniform rating system for the adherence test could be very valuable because the deviance rate reflects the efficiency of a TUB and the reasons for a deviance offer opportunities for increasing the efficiency [8]. It may be possible to introduce a common list with categories all adherence tests should be based on. Another limitation that should be mentioned is the possibility of overlooking patient-related information in the data collecting systems because of the huge amount of data. Furthermore the present study concentrated on the comparison of different tumor entities and different sized TUBs. Following studies may additionally involve the diagnosis and survival of patients related to implementation of the TUB’s recommendation. Another limiting factor in this study is the not considered influence which the physician characteristics have on the work of a TUB. It was shown due to a study from Los Angeles which investigated the influence of physician and practice characteristics on frequency of TUB attendance, that specialty and higher patient volumes are a significant predictor of more frequent attendance at a TUB [11]. This in turn is again a measure for quality of care. In the conclusion of this study tumor board agendas and formalized institution-wide policies are required to engage low-frequency attendees in order to improve quality measures.

As mentioned above deviance is a measure for the efficiency of a TUB. Different studies already dealt with factors, which have influence on the efficiency of a TUB. These include defects in the communication of doctors attending, missing information about patients´ comorbidities and their preferences, and structural and functional components [4]. These efficiency limiting factors became also clear in the present study. In order to optimize the efficiency the so called Standard Operating Procedures (SOPs) are of great importance. They represent guidelines for therapy in special tumor entities, which were created by experts and according to evidence based data. They vary in hospitals due to individual therapy concepts and procedures. In collaboration with the University Hospital Cologne the University Hospital Bonn adjusted its SOPs to individual on-site therapeutic concepts. The TUB’s therapy recommendations are based on those SOPs but personalized for each patient. By following these guidelines in choosing the right therapy recommendation a quality assurance for the patients can be done and the predefined guidelines will lead to a reduced coordination effort.

The most common cause for a deviance in the present study was the patient’s wish (36.5% of all deviances), considering that the patient’s presence at the tumorboard has been discussed a lot in past studies [13]. By patient participating at the multidisciplinary meetings the amount of inadequate therapy recommendations could be reduced, because patient’s preferences could directly be involved in the decision making and misunderstandings could be prevented. Though it must be considered that expert discussions not always can be assessed correctly by the patient [13]. A German study investigated how frequently patients are invited, which patient characteristics affect the invitation and influence on both factors due to the specific hospital. It was shown that the patient’s participation should only be performed in selected cases and the severity of the disease and the patient’s general and emotional condition needs to be evaluated individually [12].Patient’s personal attendance at the TUB could be replaced by a clarifying conversation between treating doctor and patient by reference to patient’s wishes and expectations for the forthcoming therapy, before the TUB takes place.

In 24.1% the deviance was caused by the doctor decision.72.4% of those decisions is based on changes in the progression of the patient’s disease. That means that the individual’s general condition became worse, or the patient suffered of comorbidities and side effects, which made the realization of the therapy recommendation not workable. These circumstances show a constant supervision of the therapy by the treating doctors. This point is very important, because the TUB recommendations constitute the best therapy option at a specific date, though with the possibility to adapt the treatment to changing circumstances regarding the patient’s condition. In order to reduce the amount of the left cases, which lead to deviances due to inappropriate therapy recommendations the documentation and presentation of patient-related data need to be optimized. An individually developed checklist for each TUB meeting could provide assistance to make sure that the patient cases are properly prepared before the tumorboard, but also that the interdisciplinary discussion is well structured and in this way the optimal therapy recommendation will be worked out. An example of this would be the MDT-QuIC, which was developed in the Whipps Cross University Hospital London [14].

A further important related starting point would be the optimization of documentation of patient-related data, which is the basis for the therapy recommendation made at the tumorboard. A big assistance could be a data collecting system which is not only available in one specific center, but for all doctors involved in the patient’s treatment [15]. In this way the search for documentation of past therapies could be eased and time could be saved. At the same time the number of inadequate therapy recommendations could be decreased.

In 2011 a study from Great Britain dealt with starting points for optimizing the multidisciplinary decisions made at tumorboards. Regarding this TUB members in different locations and of different specialist disciplines were interviewed. For them, the most frequent suggestions for improvement were the wish for a saved calculation of the time spent at the tumorboards as working hours, furthermore a better structured procedure in the TUBs. Important factors, which influence the decision making, were a current imaging and histopathological results, which need to be present to work out an adequate therapy recommendation [16].

## Conclusion

The decreased deviance rate from beginning of the documentation in June 2014 (10.2%) to the end of the observed period in December 2016 (6.8%) shows a positive development of efficiency in the TUBs. The number of deviances could be reduced significantly (*p* = 0.002).

By analyzing the deviances from the recommendations made at the TUBs defects could be revealed and starting points for optimizing the efficiency were found. That means the increased inclusion of patients` preferences and the attendance of selected patients at the TUB as well as the attendance of all involved practitioners and an optimized structure and presentation of patient-related data for reducing bad decisions at the TUBs. Furthermore, the introduction of a nationwide data system could ease information acquisition and could decrease the number of not assessable patient cases.

Adherence tests such as the present study consequently are the basis for the development of TUBs to further optimization of the concept of the oncological therapy. Following studies should tie onthe present study design with the aim of creating a uniform rating system concerning deviance rates. These results should be supplemented by subsequent studies by analyzing other tumor entities and adding information about diagnosis and outcome of the patients.
